# Chlorine Dioxide Is a Size-Selective Antimicrobial Agent 

**DOI:** 10.1371/journal.pone.0079157

**Published:** 2013-11-05

**Authors:** Zoltán Noszticzius, Maria Wittmann, Kristóf Kály-Kullai, Zoltán Beregvári, István Kiss, László Rosivall, János Szegedi

**Affiliations:** 1 Department of Physics, Budapest University of Technology and Economics, Budapest, Hungary; 2 Jósa András Hospital, Nyíregyháza, Hungary; 3 St. Imre Hospital, Budapest, Hungary; 4 Semmelweis University, Budapest, Hungary; University of Iowa Carver College of Medicine, United States of America

## Abstract

**Background / Aims:**

ClO_2_, the so-called “ideal biocide”, could also be applied as an antiseptic if it was understood why the solution killing microbes rapidly does not cause any harm to humans or to animals. Our aim was to find the source of that selectivity by studying its reaction-diffusion mechanism both theoretically and experimentally.

**Methods:**

ClO_2_ permeation measurements through protein membranes were performed and the time delay of ClO_2_ transport due to reaction and diffusion was determined. To calculate ClO_2_ penetration depths and estimate bacterial killing times, approximate solutions of the reaction-diffusion equation were derived. In these calculations evaporation rates of ClO_2_ were also measured and taken into account.

**Results:**

The rate law of the reaction-diffusion model predicts that the killing time is proportional to the square of the characteristic size (e.g. diameter) of a body, thus, small ones will be killed extremely fast. For example, the killing time for a bacterium is on the order of milliseconds in a 300 ppm ClO_2_ solution. Thus, a few minutes of contact time (limited by the volatility of ClO_2_) is quite enough to kill all bacteria, but short enough to keep ClO_2_ penetration into the living tissues of a greater organism safely below 0.1 mm, minimizing cytotoxic effects when applying it as an antiseptic. Additional properties of ClO_2_, advantageous for an antiseptic, are also discussed. Most importantly, that bacteria are not able to develop resistance against ClO_2_ as it reacts with biological thiols which play a vital role in all living organisms.

**Conclusion:**

Selectivity of ClO_2_ between humans and bacteria is based not on their different biochemistry, but on their different size. We hope initiating clinical applications of this promising local antiseptic.

## Introduction

The emergence and dissemination of new antibiotic-resistant bacterial strains caused by an overuse of antibiotics [1] is a global public-health concern. Methicillin Resistant Staphylococcus aureus (MRSA) [1,2] and Carbapenem- or Extreme Drug-Resistant *Acinetobacter baumannii* [3,4] are only two well known examples for such bacteria attracting world wide attention. Moreover, while the number of antibiotic resistant infections is on the rise, the number of new antibiotics is declining [1,2]. As a result of such a dangerous situation, searches for new antimicrobial agents, as well as strategies including a switch from antibiotic to antiseptic therapies, whenever that is feasible, have been initiated.

When treating local infections of wounds, ulcers or an infected mucous membrane, the application of antiseptics instead of antibiotics is a reasonable alternative especially because bacteria are less able to develop resistance against them [5]. Presently the majority of the antiseptics used for wounds [6] are organic compounds. The most frequently applied ones [6] are chlorhexidine (chlorhexidine digluconate), octenidine (octanidine dihydrochloride), polyhexanide (polyhexametylene biguanide) and triclosan (5-chlorine-2-(2,4-dichlorphenoxy)-phenol). Notable exceptions are PVP-iodine (poly(vinylpirrolidone)-iodine complex) [6] where the active ingredient is iodine, and silver [7], both being inorganic compounds.

There are some other, less used, inorganic antiseptics such as aqueous sodium hypochlorite (NaOCl), or hydrogen peroxide (H_2_O_2_) solutions, or ozone (O_3_) gas which have some applications in dentistry [8]. These compounds, however, are mainly used as disinfectants because they can be toxic even in low concentrations, a property seriously limiting their antiseptic applications. NaOCl, for example, one of the most commonly used components of irrigating solutions in endodontic practice, can cause poisoning and extensive tissue destruction if it is injected (inadvertently) into periapical tissues in the course of endodontic therapy [9]. H_2_O_2_ is also a double edged sword against bacteria as it also hurts living tissue [10]. Moreover, many bacteria are able to resist H_2_O_2_ as their catalase enzyme is able to decompose H_2_O_2_ rapidly [11]. Thus, beside toxicity, resistance can be also a problem even with the use of inorganic disinfectants [5]. It would be therefore reasonable to choose an antiseptic which would be free of such problems. We believe that in this respect chlorine dioxide (ClO_2_) may be the right choice, moreover ClO_2_ has other characteristic features favourable for antiseptic applications.

In the last twenty or more years chlorine dioxide emerged as a new and popular inorganic disinfectant. It is often referred to as „the ideal biocide” [12] because of its advantageous properties. In spite of that, as far as we know, ClO_2_ solutions are not frequently used as antiseptic. This is because the available ClO_2_ solutions were more or less contaminated with other chemicals applied in its synthesis and that contamination formed a major obstacle in medical applications like treating infected wounds, for example. Since 2006, however, with the help of an invention [13], it is relatively easy to produce high purity aqueous ClO_2_ solutions. These solutions are already commercially available [14] and have been successfully used in dentistry [15] since 2008. Thus, it seems reasonable to ask the question whether the “ideal biocide” in its pure form can also be an “ideal local antiseptic” at the same time.

Such an ideal local antiseptic should satisfy many criteria. First of all, it should be safe: it should act only locally to avoid the danger of systemic poisoning and should not inflict cytotoxic effects even in the disinfected area. In this respect, it is one of the main aims of the present work to find a reasonable answer for the following intriguing question: how is it possible that contacting or even drinking ClO_2_ solution is practically harmless for animals [16] and human beings [17], while the same aqueous solution can be a very effective and a rapid killer for bacteria, fungi, and viruses? What is the basis of this unexpected selectivity?

The answer suggested in the Results section is the following: the selectivity between humans or animals and microbes is based not on their different biochemistry, but on their different size. Denominating ClO_2_ in the title as a „size selective” antimicrobial agent aims to emphasize this new type of selectivity. To reach that conclusion, ClO_2_ transport was studied experimentally via protein membranes. The results of these experiments were evaluated applying a reaction-diffusion model for the ClO_2_ transport in a reactive medium to obtain the diffusion coefficient of ClO_2_, and the concentration of reactive groups in a protein medium. Based on these parameters the killing time, the time needed to flood a bacterium completely with ClO_2_, can be calculated. (Details of the reaction-diffusion model and the derivation of formulae estimating the killing time are given in the Information S1.) It was found that the characteristic time necessary to kill a microbe is only a few milliseconds. As ClO_2_ is a rather volatile compound its contact time (its staying on the treated surface) is limited to a few minutes. While this stay is safely long enough (being at least 3 orders of magnitude longer than the killing time) to inactivate all bacteria on the surface of the organism, it is too short for ClO_2_ to penetrate deeper than few tenths of a millimetre; thus, it cannot cause any real harm to an organism which is much larger than a bacterium.

In the Discussion part, it is shown that ClO_2_ can meet the safety and effectiveness requirements for a local antiseptic. Next, the chemical mechanism of the antiseptic action of ClO_2_ is discussed and compared with that of hypochlorous and hypoiodous acids (HOCl and HOI) which are „natural” antiseptics. These hypohalous acids are used by neutrophil granulocytes, the most abundant type of white blood cells in mammals, to kill bacteria after phagocytosis. Both hypohalous acids and also ClO_2_ attack sulfhydryl groups [18,19] which play an essential role in the life processes of all living systems, e.g. in ATP synthesis. That explains why bacteria were not able to develop resistance against HOCl during eons of evolution and why the emergence of ClO_2_ resistant bacterial strains cannot be expected either. Besides this similarity, however, there are also important dissimilarities among these reagents, e.g. ClO_2_ is more selective than HOCl. Last of all, circulation in multicellular organisms can provide some additional protection to these organisms against ClO_2_.

## Methods

### Materials

Reagent grade chemicals were purchased from Sigma-Aldrich and pork skin gelatine from Fluka (48719). High purity chlorine dioxide solutions were produced according to our invention [13]. Dried pig bladders were purchased in the Great Market Hall of Budapest at the shop “Solvent” (www.solvent.hu). These bladders are usually applied for kulen sausage production. 

### Physico-chemical methods

#### Measurement of ClO_2_ permeation through protein membranes

**Figure 1:**
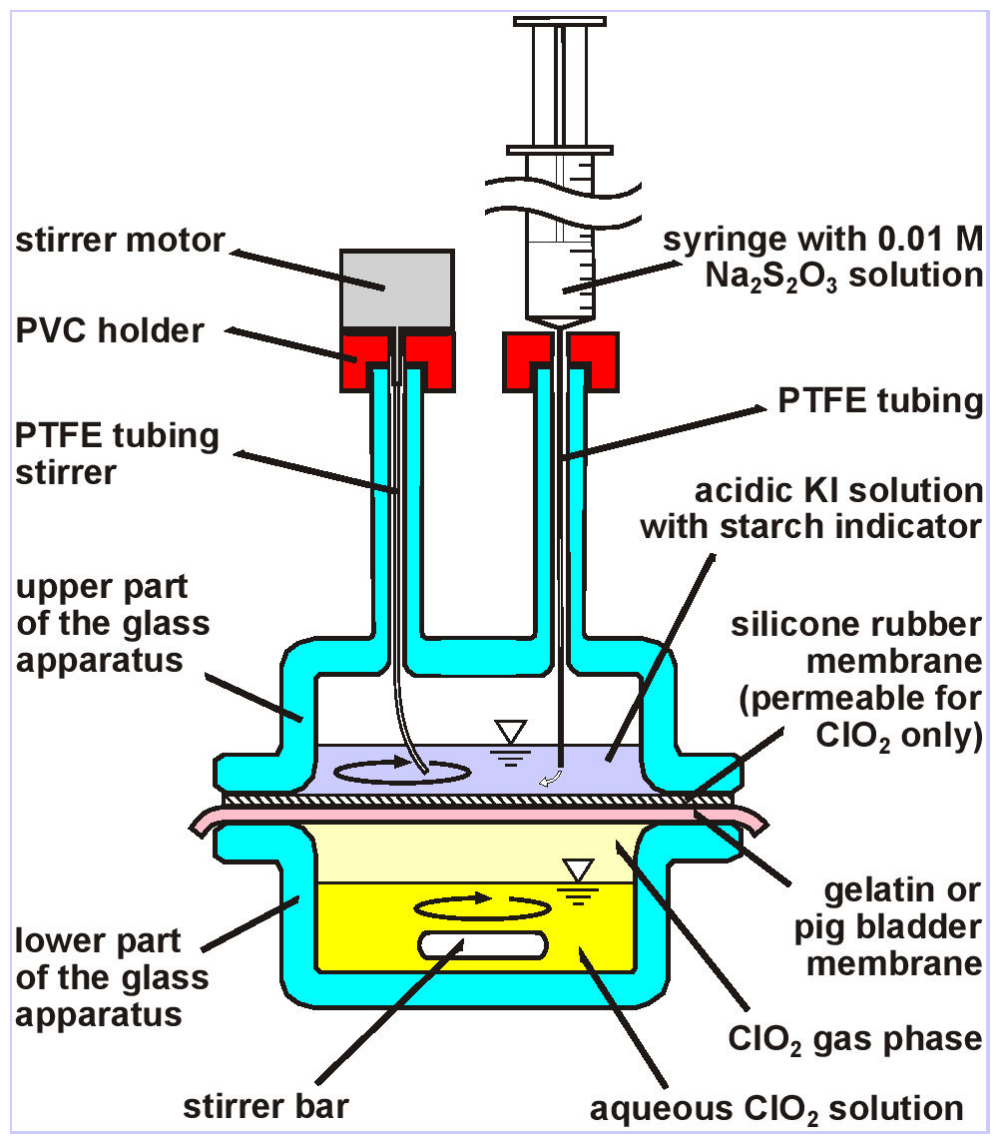
Apparatus to measure ClO_2_ transport through gelatine or pig bladder membranes. The two glass parts of the apparatus are held together by a pair of extension clamps (not shown in the Figure) which are fixed to a support stand by clamp holders. The active cross-section of the membranes is 28 cm^2^. See text for the working principle.

The rate of ClO_2_ transport was measured with the apparatus shown in Figure 1 through two kinds of protein membranes: gelatin and pig bladder membranes, respectively. Choosing a membrane geometry for the experiments is advantageous because then the problem is „one dimensional”, the concentration is a function of only one spatial coordinate *x*, which is perpendicular to the membrane, and the concentration distribution can be given as *c*=*c*(*x,t*)*.*

As Figure 1 shows, the membrane is in a horizontal position and the transport of ClO_2_ takes place across the membrane bounded by two horizontal planes we denote by *x*=0 and *x*=*d* in our calculations, where *d* is the thickness of the membrane.

Constant ClO_2_ concentrations are maintained at both boundaries of the membrane, i.e. we have constant boundary conditions: c(*0,t*)*=c*_*0*_ and *c*(*d,t*)*=0*, respectively. There is no ClO_2_ in the membrane at the start of the experiment, so the initial condition: *c*(*0<x≤d,0*)*=0* (see Figure S1 in Information S1).

While the lower face of the protein membrane is not in direct contact with the liquid phase, such direct contact would not make any difference regarding the ClO_2_ transport. This is because the chemical potential of ClO_2_ in the liquid and the vapour phase is the same due to the equilibrium between the liquid and the vapour phase established by continuous stirring.

Above the protein membrane there is a silicone rubber membrane in order to block the transport of any other chemicals except ClO_2_. Silicone rubber is highly permeable for chlorine dioxide, but it is practically impermeable for other reagents [13]. This way the ClO_2_ transport across the test membrane can be measured selectively.

Both protein membranes had a thickness of 0.5 mm and a diameter of 10 cm. The diameter of the active area in the apparatus was 6 cm resulting in an active area of 28 cm^2^. The volume of the aqueous ClO_2_ solution was 40 ml and its ClO_2_ concentration was around 1000 ppm. (The exact value is given at each experiment.)

After crossing the membranes, ClO_2_ enters the upper aqueous solution which is made by mixing 10 ml of water, 2 ml of 1 M sulphuric acid, 1 ml of 1 M KI, and 0.5 ml of 0.01 M Na_2_S_2_O_3_ and as an indicator, two drops of 5 % starch solution is also added. When ClO_2_ enters the upper solution, it oxidizes iodide to iodine, which, in turn, is reduced back to iodide again by Na_2_S_2_O_3_ as long as thiosulphate is in excess. However, when all thiosulphate is consumed, the intense blue-black colour of the starch-triiodide complex appears suddenly. The time *t* when the whole solution becomes homogeneously black (the time of the „black burst”) was recorded and another 0.5 ml of Na_2_S_2_O_3_ solution was added with the help of the syringe shown in the Figure. Addition of the thiosulphate eliminated the blue-black colour immediately but, after a certain period, when enough new ClO_2_ was transported across the membrane, it reappeared again. Then the cycle was repeated starting with the injection of a new 0.5 ml portion of the Na_2_S_2_O_3_ solution. The results of the measurements were depicted in a *V=V*(*t*) diagram where *t* is the time of the n-th dark burst and *V* = *n*×0.5 ml that is the total volume of the thiosulphate solution added before the *n*-th breakthrough.

The experiments were performed at laboratory temperature 24 ± 2 °C.

### Preparation of the gelatin membrane

To prepare a mechanically strong membrane, it was reinforced by filter paper and the gelatin was cross-linked with glutaraldehyde. As the cellulose in the filter paper does not react with ClO_2_ from the point of our experiments, it is an inert material.

10 ml of 10 % aqueous gelatin solution was mixed rapidly with 0.5 ml of 25 % glutaraldehyde solution at room temperature, and a filter paper disk (diameter: 10 cm) was soaked with the mixture. Then the disk was placed between two glass plates covered with polyethylene foils. Spacers were applied to produce a 0.5 mm thick membrane. After a 2 hour setting time the filter paper reinforced gelatin membrane was removed from the form and it was placed into distilled water overnight before the measurements.

### Preparation of the pig bladder membrane

For the experiments, membrane disks with 10 cm diameters were cut from commercially available pig bladders and they were kept in distilled water for one day at +4 °C to stabilize their water content. The pig bladder membranes are slightly asymmetric: the surface of one side is smoother than the other. To obtain reproducible results, the membrane was always fixed in the apparatus with its smoother side facing downwards.

## Results

Our results cover the following themes: First, we present and evaluate membrane transport experiments aiming to determine

i) the diffusion coefficient of ClO2 D in a reactive protein medium, and
ii) the concentration of reactive groups s0 in that medium.

To evaluate the membrane transport experiments we applied a reaction-diffusion model for the transport of ClO_2_ in a medium containing reactive proteins. The details of that theory and the mathematical derivation of formulas applied in this section are given in the Information S1. Then, based on the experimentally determined *D* and *s*_*0*_ we calculate *T*_*KILL*_, the time needed to kill bacteria by ClO_2_, and *p*, the penetration depth of ClO_2_ into human tissue during a wound healing treatment. 

ClO_2_ permeation was measured via gelatin and pig bladder membranes. The apparatus is shown in Figure 1 of the Methods section.

### Permeation of ClO_2_ through an artificial gelatin membrane

Gelatin was our first choice for a model material because we wanted to study the ClO_2_ transport in a protein medium with a known amino acid composition. Pork skin gelatin (Fluka 48719) contains only two amino acids that can react with ClO_2_: methionine (0.88 %) and tyrosine (0.6 %) [20].

**Figure 2:**
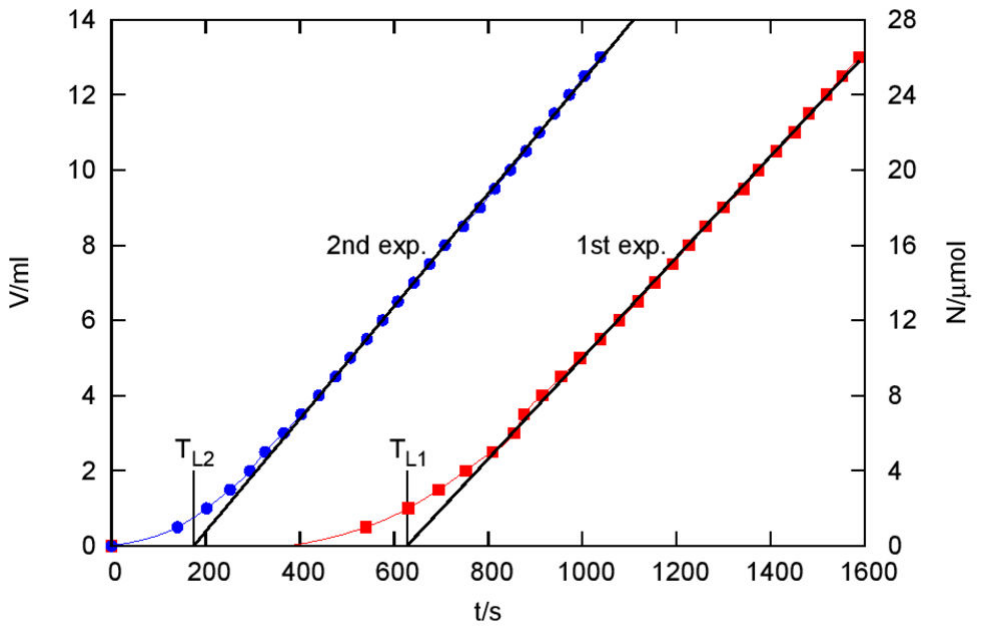
Permeation of ClO_2_ through a gelatin membrane as a function of time *t*. Each point in the diagram represents a „black burst” (see Methods). *V* is the cumulative volume of the 0.01 M Na_2_S_2_O_3_ titrant added before the burst and *N* is the amount of ClO_2_ permeated until time *t*. *T*_*L1*_ = 627 s and *T*_*L2*_ = 175 s are time lags of the first and the second experiments, respectively. The concentration of ClO_2_ source in the magnetically stirred aqueous solution was 1360 ppm (mg/kg) or 20.1 mM.

Figure 2 shows the results of two consecutive experiments performed with the same gelatin membrane (see the two curves denoted as 1st exp. and 2nd exp.). After the first experiment, the membrane was removed from the apparatus and was kept in distilled water for 1 hour before the second experiment.

### Calculating the ClO_2_ diffusion coefficient *D* and the effective concentration of ClO_2_ consuming substrates *s*_*0*_ in gelatin

Figure 2 shows N, the cumulated amount of ClO2 permeated through the membrane as a function of time. 

(N was calculated from the titrant volumes V that are given in Table S1 in Information S1. together with the times t of addition.) It is a common feature of both curves shown in Figure 2 that two characteristically different dynamical regimes can be observed. In the first regime, the amount of the permeated ClO2 is very small, then, after a rapid transition period the cumulated amount of ClO2 increases linearly with time. Real dynamics can be approximated with the following simplified model: zero permeation is assumed at the beginning during a waiting period but right after that a constant diffusion current appears, thus, the permeated amount increases linearly with time. To characterize such a dynamic behaviour the concept of „time lag” can be introduced: it is the time where the asymptote of the linear regime crosses the time axis [21].

Regarding the asymptotes of the corresponding curves, the time lag in the first and in the second experiment is *T*_*L1*_ = 627 s and *T*_*L2*_ = 175 s, respectively. A logical explanation for this difference is that some ClO_2_ is consumed inside the gelatin in the rapid reaction with methionine and tyrosine. So ClO_2_ can break through only after it eliminates all these highly reactive amino acid residues. In the case of the second experiment, the breakthrough occurs earlier as most of these residues already reacted with ClO_2_ during the first experiment.

If we assume that in the second experiment the reaction plays a minor role only, then in that case, the time lag is entirely due to diffusion. Roughly speaking the diffusional time lag is the time necessary to establish a steady state concentration profile inside the membrane that is to “fill up” the membrane with ClO_2_. Based on dimensional analysis considerations (the dimension of the diffusion coefficient is (length)^2^/(time) ) we can expect that the time lag should be proportional with the square of the thickness and inversely proportional with the diffusional coefficient. Really, the exact result [21] is that the diffusional time lag *T*_*DM*_ for a membrane of thickness *d* can be calculated as:

$$T_{DM}=\frac{1}{6}\cdot \frac{d^{2}}{D}$$ (1)

Thus, with the assumption *T*_*L2*_ = *T*_*DM*_ = 175 s, *D*, the diffusion coefficient of ClO_2_ in the gelatin membrane can be calculated knowing that d = 0.5 mm. The result: *D* = 2.4×10^-6^ cm^2^s^-1^.

*D* can be determined in another way as well, from the steady state regime. The steady state ClO_2_ current is the slope of the curve in the linear regime. For the 2nd experiment *J*_*2*_ = 30 nmol/s. Then Fick’s law of diffusion

$$J=A\cdot D\cdot \frac{\Delta c}{d}$$ (2)

can be applied to calculate D. Here *A*= 28.3 cm^2^ is the active cross-section of the membrane and *Δc* is the concentration difference between the two sides of the membrane. Regarding our boundary conditions *Δc*=*c*_*0*_ = 20.1×10^-3^ M. This way *D* = 2.6×10^-6^ cm^2^s^-1^ is obtained.

The two *D* values, the one calculated from the time lag and the other calculated from the steady state, agree reasonably well indicating that indeed the 175 s time lag is caused mostly by diffusion and any delay due to chemical reactions is negligible in the second experiment.

On the other hand, in the first experiment, the time lag *T*_*RM*_ is caused mostly by the reaction between ClO_2_ and the reactive amino acid residues (in short “substrates”) in the membrane. It is important to realize that *T*_*RM*_ is not due to a slowness of the reaction kinetics (as the rate constants of the relevant ClO_2_ – amino acid reactions are relatively high [22–24]), but it is due to the actual ClO_2_ consumption by the reactions within the membrane delaying the breakthrough. If we assume that the rate of the chemical reaction is limited by the diffusional transport of ClO_2_ across a zone already without reactive amino acids toward a zone of unreacted ones, then a sharp reaction front will develop on the boundary of the two zones (see Figure S1 in Information S1). The front starting from one side of the membrane and driven by diffusion, propagates slowly through the membrane and *T*_*RM*_ is the time when it arrives to the other side of the membrane. According to a detailed derivation in the Information S1, *T*_*RM*_ can be given by the so-called parabolic rate law (see equation (S12) in Information S1):

$$T_{RM}=\frac{1}{2}\cdot \frac{s_{0}}{c_{0}}\cdot \frac{d^{2}}{D}$$ (3)

where *s*_*0*_ is the initial effective substrate concentration, i.e. the ClO_2_ consuming capacity of the membrane in unit volume, and *c*_*0*_ is ClO_2_ concentration at the boundary of the membrane.

Substituting the more reliable diffusion coefficient measured in the steady-state of the second experiment *D* = 2.6×10^-6^ cm^2^s^-1^ and applying the assumption that *T*_*RM*_ = *T*_*L1*_ = 627 s, the effective substrate concentration of the gelatin membrane *s*_*0*_ can be calculated. The result: *s*_*0*_ = 26.2 mM.

### Permeation of ClO_2_ through a pig bladder membrane

**Figure 3:**
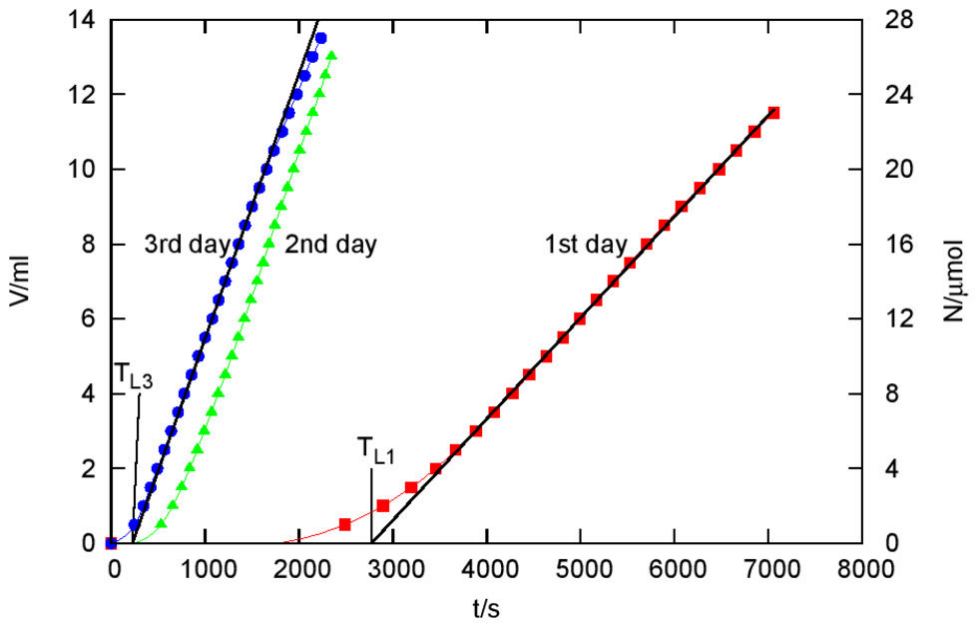
Permeation of ClO_2_ through a pig bladder membrane as a function of time *t*. *V* and *N* have the same meaning like in Figure 2. *T*_*L1*_ = 2770 s, *T*_*L2*_ = 586 s and *T*_*L3*_ = 226 s are time lags of the experiments performed on the 1st, 2nd, and 3rd day, respectively. The concentration of the ClO_2_ source was 946 ppm (14.0 mM) in these experiments.

In this experiment, we studied the ClO_2_ permeability of a pig bladder membrane which is a relatively thin (in our case it was 0.5 mm thick) but sturdy animal tissue. The same apparatus was applied as in the case of the gelatin membrane and the experimental points were depicted in Figure 3 also with the same method. (Titrant volumes and the time of addition are given in Table S2 in Information S1.) 

All the three measurements (indicated as 1st day, 2nd day and 3rd day) were performed with the same pig bladder membrane but on three successive days. The membrane was kept in distilled water at +4 °C overnight between the experiments which were always started with fresh solutions.

To check the reproducibility of our measurements, we repeated the measurements with another pig bladder membrane (not shown in the Figure). While the new membrane was from a different pig bladder and its blood vessel pattern was also different, the relative deviation between the results of the two series of experiments was surprisingly small: only about 10 %. (The blood vessel structure of the membrane becomes visible as a dark network before a „black burst” because the permeability of the membrane is somewhat higher through those vessels.)

Another interesting observation was that the pig bladder membrane maintained its integrity and its mechanical strength even after the third experiment. This is because ClO_2_ reacts selectively with certain amino acid residues of the proteins but does not destroy the peptide bonds thus the primary protein structure can survive.

### Calculating the ClO_2_ diffusion coefficient and the effective concentration of ClO_2_ consuming substrates in pig bladder

Evaluation of the results was made in a similar way as in the case of the gelatin membrane. It was assumed that the time lag measured in the third experiment *T*_*L3*_ =226 s is a purely diffusional time lag that is *T*_*L3*_ ≈ *T*_*DM*_. The diffusion coefficient of ClO_2_ in a pig bladder membrane calculated from the above assumption is *D* = 1.84×10^-6^ cm^2^s^-1^. That value is in good agreement with the *D* = 1.80×10^-6^ cm^2^s^-1^ value calculated from the steady state current *J*_*3*_ = 14.1 nmol/s of the 3rd experiment.

As we can see, the diffusion coefficient of ClO_2_ in a pig bladder tissue is only 30 % smaller than in the unstructured gelatin. This supports our assumption that the cellular structure of the pig bladder tissue does not matter too much from the point of the diffusional transport of ClO_2_ as it can penetrate through the external and internal lipid membranes of the individual cells of the tissue.

However, there is a more significant deviation between the pig bladder and the gelatin regarding *s*_*0*_, the effective substrate concentration. Assuming that the time lag in the first experiment *T*_*L1*_ = 2770 s is due to the chemical reaction, *T*_*L1*_ ≈ *T*_*RM*_, then from (3) we get *s*_*0*_ = 56 mM, indicating that the concentration of the reactive components in the pig bladder tissue is about two times higher than that is in the gelatin. This is a reasonable result as the animal tissue is denser, and it contains not only methionine and tyrosine like gelatin, but also cysteine and tryptophan residues.

We would like to add that in a series of measurements performed with the same membrane the steady state ClO_2_ current in the first experiment is always smaller than in the subsequent ones, although the ClO_2_ source is not changed. This effect is more pronounced in the case of an animal membrane (compare the slope of the 1st day experiment with that of the other days). The phenomenon can be understood if we assume that some components, which are able to react with ClO_2_ but only slowly, can remain in the pig bladder even after the first ClO_2_ breakthrough. As it is shown in equation (S40) in the Information S1, the slow ClO_2_ consumption of these components can explain a smaller quasi-steady state current. The fact that these components disappear from the membrane after keeping it in water overnight suggests that they are reaction products which can be leached out from the membrane or are unstable intermediates which decompose.

### Estimating the killing time for bacteria with cylindrical and spherical geometries

We assume that a bacterium is killed when its whole volume is flooded by ClO_2_. To calculate the killing time, if we know the shape and the size of the bacterium, we would need two more parameters, the diffusion coefficient of ClO_2_ *D* and the effective concentration of ClO_2_ consuming substrates *s*_*0*_ in the bacterial medium. In the absence of bacterial data it will be assumed that the parameters *D* and *s*_*0*_ in the single cell of a bacterium are close to that what we have measured above in the animal cell aggregates of the pig bladder. A further simplifying assumption is that only spherical and cylindrical bacteria are considered. Numerical results are calculated for a diameter of 1 μm, which is a characteristic length-scale for bacteria. Mathematical formulas for the killing time and the penetration depth are derived in the Information S1. In this section only the results of those derivations will be given together with some qualitative explanations on their meaning.

It will be assumed that the rate of the “ClO_2_ – bacterium reaction” is also limited by the diffusion of ClO_2_ to the fast reacting amino acid residues fixed in protein molecules like in the case of the much larger membranes and this way a sharp reaction front propagates from the cell wall toward the centre of the bacterium.

Intuitively, the killing time T_*KILL*_ should be analogous to the time lag *T*_*RM*_ in a membrane caused by a chemical reaction, because these are the times needed to flood the whole volume. We can expect, however, that the geometric factor should be different depending on the shape of the bacterium. For a cylindrical bacterium with a diameter of *d* the killing time is

$$T_{KILL,C}=\frac{1}{16}\cdot \frac{s_{0}}{c_{0}}\cdot \frac{d^{2}}{D}$$ (4)

see equation (S18) in the Information S1, and for a spherical bacterium also with a diameter of *d* it is

$$T_{KILL,S}=\frac{1}{24}\cdot \frac{s_{0}}{c_{0}}\cdot \frac{d^{2}}{D}$$ (5)

according to equation (S24) in Information S1. We can see that (4) and (5) are analogous to (3) but the geometric factors for a cylinder and for a sphere are much smaller than for the planar membrane indicating that in these geometries the surface from where diffusion current is starting is relatively larger compared to the volume that has to be flooded.

Substituting the pig bladder parameters *D* = 1.8×10^-6^ cm^2^s^-1^ and *s*_*0*_ = 56 mM into formulas (4,5) together with the ClO_2_ concentration applied in the wound healing experiments (see later) *c*_*0*_ = 4.45 mM (Solumium Oral^®^, 300 ppm) and using d = 1 μm we obtain that the killing time for a cylindrical bacterium with a diameter of 1 μm is

$$T_{KILL,C}=4.4\text{ ms,}$$

while the killing time for a spherical bacterium with a diameter of 1 μm is

$$T_{KILL,S}=2.9\text{ ms}.$$

As we can see, the killing time for a bacterium is only a few ms due to its small size. Even if *s*_*0*_, the effective substrate concentration of a bacterium would be an order of magnitude higher than we assumed, the killing time would be still less than 0.1 s. Other approximations applied in our calculations can only overestimate the real killing time. For example, the diffusion coefficient of ClO_2_ in the pig bladder was measured at 24 ± 2 °C. If ClO_2_ is used to disinfect a living human tissue, the temperature is higher, which means a larger diffusion coefficient and an even shorter killing time. Another approximation is the concept of fixed substrates. Inside a bacterium mobile substrates like glutathione [25], free amino acids and various antioxidants also occur. These small molecules can diffuse by and large freely within the bacterium. Nevertheless *T*_*KILL*_ would still work as a good upper estimate because the mobility of the substrate can only shorten the time needed for ClO_2_ to reach these substrates and react with them. Furthermore, when the killing time T_*KILL*_ is regarded as the time when the sharp front reaches the center of the sphere or the symmetry axis of the cylindrical bacterium, it will surely be overestimated, as it is not necessary to oxidize all the available substrate content of a bacterium to kill it. For example, it is enough to oxidize less than 40 % of the methionine content of *E. coli* to achieve a 100 % kill [26].

### Contact time and penetration depth of ClO_2_ into human skin or wound

When an organism is not submerged in the aqueous ClO_2_ solution but the solution is applied on its surface only, as in the case of disinfecting wounds, the volatility of ClO_2_ also has to be taken into account. The effective contact time is much shorter using a ClO_2_ solution than with less or non-volatile disinfectants. According to our measurements, when a wound is covered with 3 wet and 3 dry layers of gauze more than 80 % of ClO_2_ evaporates from the bandage within one minute due to the high volatility of ClO_2_ and to the high specific surface of the gauze. Thus, to give an upper limit for the penetration depth into the human tissue, we will assume that the initial ClO_2_ concentration (*c*_*0*_ = 4.45 mM, Solumium Oral^®^) is maintained for 60 s, that is *T*_*CON*_ = 60 s, where *T*_*CON*_ denotes the contact time. As a zero-th estimate, we assume again that the human tissue has the same *D* and *s*_*0*_ values like that of the pig bladder tissue.

Applying the parabolic rate law (see equation (S13) in Information S1 where *t* = *T*_*CON*_) the penetration depth *p* can be estimated:

$$p=\sqrt{\frac{2c_{0}D\cdot T_{CON}}{s_{0}}}$$ (6)

*p*(*T*_*CON*_ = 60 s) = 41.5 μm. We remark that (6) can be derived from (3) directly if we realize that for the present problem d = *p* and *T*_*RM*_ = *T*_*CON*_.

Nevertheless, the actual penetration depth into a living tissue - either its surface is a wound or an intact human skin – should be even much smaller than the above estimate. This is due to the capillary circulation which is present in living tissue but is absent from dead tissue like the pig bladder membrane used for the measurements. The serum in the blood vessels and also the extracellular fluid contain many components capable of reacting rapidly with ClO_2_. The fluid transport of these reactive components in the blood capillaries of the dermis [27] can maintain a finite reactant concentration in that region. Then the diffusive transport of these reactants outward from the dermis into the epidermis [27] can halt an inward propagating reaction front establishing a steady state.

Moreover, in the case of intact human skin, ClO_2_ should permeate through the stratum corneum [28] first, which is the 10–40 μm thick outermost layer of epidermis consisting of several layers of dead cells. This keratinous layer forms a barrier to protect the underlying tissue from infection, dehydration and chemicals. The diffusion coefficient of ClO_2_ in that layer should be much lower compared to the underlying tissue.

As we can see, the penetration depth into human skin is only few tens of a micrometer even if we neglect circulation. Such shallow penetration cannot really harm human tissues. On the other hand, this short contact time is still several orders of magnitude larger than the killing time, *T*_*CON*_ >> *T*_*KILL*_, which is the necessary criterion of a successful disinfection.

### Therapeutic window

The above formulas and calculations indicate that disinfection of living tissues with aqueous ClO_2_ solutions has a very wide therapeutic window: while surprisingly low concentrations and short contact times are able to kill bacteria, much higher concentrations and residence times are still safe to use.

There is one notable exception: inhaling high concentration ClO_2_ gases for an extended time can be dangerous for human health because the alveolar membrane is extremely thin (a mere 1-2 microns and in some places even below 1 micron). The effect of ClO_2_ in these membranes is somewhat counterbalanced, however, by the intense blood circulation there.

## Discussion

In this section first we discuss whether ClO_2_ should be regarded as an “exotic” antiseptic only or it has the promise to become a commonly used antiseptic to treat local infections. To this end safety and effectiveness requirements for a local antiseptic are collected to check how ClO_2_ can meet these requirements compared to other antiseptics.

Next a biochemical action mechanism, explaining the antiseptic effect of ClO_2_ is discussed, which is partly analogous to that of hypochlorous and hypoiodous acids. These “natural” antiseptics also react, among others, with sulfhydryl groups like ClO_2_ but their reaction products can be different. The importance of that difference and the protective role of SH groups and of the circulatory system, existing in a multicellular organism only, is also discussed.

### Safety and effectiveness requirements for a local antiseptic

A local antiseptic should meet the following requirements to be considered as safe:

i) it should act only locally to avoid systemic poisoning, and
ii) it should not prevent or delay the process of healing, i.e. it should not be cytotoxic.

and as effective:

iii) it should be effectual in relatively low concentrations, and even in biofilms (biofilms are medically important, accounting for over 80 percent of microbial infections in the body [29]) as well, and
iv) microbes should not be able to develop resistance against it (a problem related to the biochemical mechanism of action).

As it was shown in the Results section ClO_2_ as a size selective antiseptic, meets requirements i) and ii). Thus only criteria iii) and iv) are discussed here.

### Comparing the biocidal activity of ClO_2_ to that of other antiseptics (criterion iii)

In free aqueous solutions, the strongest chemical disinfectant is ozone. In biofilms, however, the performance of ozone is rather poor. In addition, ozone is toxic and decomposes in aqueous solutions rapidly. (Its half life is only 15 min at 25 °C at pH 7.) All of these disadvantageous properties of ozone prevent its use as an antiseptic in most applications.

The second strongest disinfectant after ozone is chlorine dioxide. Tanner [30] made a comparative testing of eleven disinfectants on three test organisms (including two bacteria: Staphylococcus aureus and Pseudomonas aeruginosa and one yeast: Saccharomyces cerevisiae). He found that the disinfectant containing ClO_2_ had the highest biocidal activity on a mg/l basis against the test organisms. Beside antibacterial and antifungal properties, ClO_2_ also shows strong antiviral activity, about ten times higher than that of sodium hypochlorite [31]. And it inactivates practically all microbes including algae and animal planktons [32] and protozoans [33].

Moreover ClO_2_ can remove biofilms swiftly [12] because it is highly soluble in water and unlike ozone it does not react with the extracellular polysaccharides of the biofilm. This way ClO_2_ can penetrate into biofilms rapidly to reach and kill the microbes living within the film.

### Impossibility of bacterial resistance against ClO_2_ (criterion iv)

ClO_2_ is a strong, but a rather selective oxidizer. Unlike other oxidants it does not react (or reacts extremely slowly) with most organic compounds of a living tissue. ClO_2_ reacts rather fast, however, with cysteine [22] and methionine [34] (two sulphur containing amino acids), with tyrosine [23] and tryptophan [24] (two aromatic amino acids) and with two inorganic ions: Fe^2+^ and Mn^2+^. It is generally assumed that the antimicrobial effect of ClO_2_ is due mostly to its reactions with the previously mentioned four amino acids and their residues in proteins and peptides. In the peptide group it is important to mention glutathione – a small tripeptide containing cysteine – which is a major antioxidant in cells, with an intracellular concentration of 0.1-10 mM [35].

Margerum’s group [22–24] reported the following second order rate constants at pH 7 and 25 °C: cysteine 1×10^7^ M^-1^s^-1^ >> tyrosine 1.8×10^5^ M^-1^s^-1^ > tryptophan 3.4×10^4^ M^-1^s^-1^. As can be seen, cysteine is the far most reactive amino acid because of its thiol group. As the above mentioned four amino acids and especially cysteine and biological thiols play a crucial role in all living systems, including microbes, it is impossible for any microbe to develop a resistance against chlorine dioxide.

As an important analogy we can mention that bacteria have never been able to become resistant against hypochlorous acid (HOCl) either, which is an important natural antiseptic used by neutrophils for millions of years. Neutrophils, a type of white blood cells, are phagocytes which kill the engulfed microbes by applying various hydrolytic enzymes and hypohalogeneous acids, chiefly HOCl [36,37]. On that basis Robson and co-workers applied HOCl as a kind of „natural” wound care agent [38,39]. Thus, it is reasonable to compare the action mechanisms and other properties of ClO_2_ and HOCl as antiseptic agents.

### Comparison of ClO_2_ and HOCl as possible antiseptic agents

HOCl, like ClO_2_, reacts rapidly with the sulphur containing amino acid residues of methionine and cysteine, the second order rate constant (at pH 7.4 and 22 °C) being 3.8×10^7^ M^-1^s^-1^ and 3.0×10^7^ M^-1^s^-1^, respectively, and also reacts with tryptophan (1.1×10^4^ M^-1^s^-1^) and tyrosine (44 M^-1^s^-1^) [40]. However, unlike ClO_2_, HOCl reacts rapidly with many other amino acid residues and even with peptide bonds [40], and many other compounds such as carbohydrates, lipids, nucleobases, and amines [41].

As we can see the important similarity is the fast reaction of both HOCl and ClO_2_ with the SH group of cysteine. This is important because it is assumed that abolition of ATP synthesis and killing bacteria by HOCl is due to its reaction with sulfhydryl groups [18]. It is a logical assumption that ClO_2_ can also stop the ATP synthesis as it reacts with the very same SH groups like HOCl.

At the same time, however, there are important dissimilarities between HOCl and ClO_2_:

i) HOCl is much less specific and reacts rapidly with numerous other substrates. Thus killing bacteria with HOCl requires more reagent than with ClO_2_.
ii) While ClO_2_ evaporates rapidly from its aqueous solution and can reach and kill bacteria even through a gas phase, e.g. through an air bubble blocking a dental root canal [42], evaporation of HOCl is not significant. Thus HOCl stays at the disinfected area for a long time even after killing all bacteria which can cause inflammation there [43].
iii) HOCl is a more drastic reagent and causes irreversible damage. For example ClO_2_ oxidizes glutathione (GSH) mainly to glutathione disulfide (GSSG) [22] which can be reduced back to GSH easily in a natural way in the body. On the other hand, HOCl can attack disulfide bonds and oxidizes GSH mostly to glutathione sulfonamide (GSA) [44] causing an irreversible loss of the cellular GSH.

### Sulfhydryl groups and circulation can protect multicellular organisms from ClO_2_ inflicted irreversible damage

As it was mentioned, the ClO_2_ –SH group reaction has the highest rate constant among the ClO_2_ – amino acid reactions. (Cysteine or GSH [22] reacts about 50 times faster than the runner up tyrosine.) Consequently, as long as some SH groups are present (mostly in the form of GSH), these groups react with ClO_2_ rapidly protecting other amino acid residues from oxidative damage. Moreover the oxidation of SH groups to disulfide bonds can be reversed. An interesting example was presented by Müller and Kramer [45,46]. They found that the cytotoxic effect of povidone-iodine after a 30 min contact with murine fibroblast was only temporal: after a 24 hour culture without the antiseptic an unexpected revitalization of the fibroblasts was observed [45]. According to Winterbourn and co-workers [47], HOI (the reactive hydrolysis product of iodine) also oxidises GSH to GSSG but not to GSA. That parallelism between the reversible HOI-GSH and the ClO_2_-GSH reactions raises the question whether an analogous revitalization might be also possible in the case of ClO_2_. This question is all the more justified since in some animal experiments [16] rats were drinking water containing 200 ppm ClO_2_ for 90 days but without developing any gastrointestinal problems. In those experiments all ClO_2_ must have reacted with the animal tissues as it cannot evaporate from the stomach of the rats. To interpret that result it is reasonable to assume that SH groups transported by the circulation system of the rodent protected the epithelial cells in its gastrointestinal tract from an irreversible oxidation by ClO_2_.

Above a certain limit, however, when a too high percentage of the protective SH groups is already oxidized, ClO_2_ would inflict irreversible changes to the higher order protein structures by oxidizing the tyrosine and tryptophan residues [48]. That would certainly happen with the bacteria on the surface of an infected tissue as their GSH supply [26] can be rapidly exhausted by ClO_2_. Mammalian cells below the surface, however, might survive being supported by the circulation which transports protective sulfhydryl and other reductive compounds to the cells, continuously repairing or even revitalizing them.

Thus beside their size there is another important difference between single cell and more complex multicellular organisms: it is the circulation which can help the cells of a multicellular organism to survive while that type of help is not available for a bacterium.

## Conclusion

Chlorine dioxide is a size selective antimicrobial agent which can kill micron sized organisms rapidly but cannot make real harm to much larger organisms like animals or humans as it is not able to penetrate deeply into their living tissues. Moreover the circulation of multicellular organisms can provide an additional protection to these organisms against ClO_2_.

It is an aim of the present work to initiate clinical studies hoping that ClO_2_ could be applied to treat various local infections, especially where bacterial resistance is a problem. We have already obtained an official permission [49] to start such studies.

## Supporting Information

Information S1 **This file contains the description of a reaction-diffusion (RD) model for the transport of ClO_2_ in a medium containing reactive proteins, and its quasi steady state solution when the ClO_2_ – substrate reaction is fast and when it is slow.** Figure S1. Schematic ClO_2_ and substrate concentration profiles in a hydrogel slab. Table S1. Data depicted in Figure 2 (the cumulative volume V of the 0.01 M Na_2_S_2_O_3_ titrant added until time *t*). Table S2. Data depicted in Figure 3 (the cumulative volume V of the 0.01 M Na_2_S_2_O_3_ titrant added until time *t*).
(PDF)
