# Supplementary material for: Chlorine Dioxide Is a Size-Selective Antimicrobial Agent
Source: PLoS One. 2013 Nov 5;8(11):e79157. doi: 10.1371/journal.pone.0079157 (PMC3818415; doi:10.1371/journal.pone.0079157)
Supplement: Information S1 — This file contains the description of a reaction-diffusion (RD) model for the transport of ClO2 in a medium containing reactive proteins, and its quasi steady state solution when the ClO2 – substrate reaction is fast and when it is slow. Figure S1. Schematic ClO2 and substrate concentration profiles in a hydrogel slab. Table S1. Data depicted in Figure 2 (the cumulative volume V of the 0.01 M Na2S2O3 titrant added until time t). Table S2. Data depicted in Figure 3 (the cumulative volume V of the 0.01 M Na2S2O3 titrant added until time t). (PDF) [file pone.0079157.s001.pdf]

Supporting Information

Chlorine dioxide is a size-selective antimicrobial agent

Zoltán Noszticzius[1], Maria Wittmann[1], Kristóf Kály-Kullai[1]
Zoltán Beregvári[2], István Kiss[3], László Rosivall[4] and János Szegedi[2]

[1]Department of Physics,
Budapest University of Technology and Economics, Budapest, Hungary
[2]Jósa András Hospital, Nyíregyháza, Hungary
[3]St. Imre Hospital, Budapest, Hungary
[4]Semmelweis University, Budapest, Hungary

A reaction-diffusion (RD) model for the transport of ClO_2 in a medium containing reactive proteins

A general RD equation for ClO_2

The following partial differential equation (usually called reaction-diffusion equation [1]) holds for the local ClO_2 concentration c (c is a function of the time t and of the space coordinates) when ClO_2 diffuses through a medium containing various components which can react with it:

$$\frac{\partial c}{\partial t} = -\sum_{i=1}^{N} R_i + D\nabla^2 c\,. \qquad \text{(S1)}$$

In equation (S1) $\frac{\partial c}{\partial t}$ is the time derivative of the local ClO_2 concentration, R_i is the rate of the ClO_2 consumption due to the i-th reaction at the same location, N is the number of the various ClO_2 consuming reactions, D is the diffusion coefficient of ClO_2 in the medium, and $\nabla^2 c$ is the Laplacian of c, which, applying a three dimensional Descartes coordinate system with spatial coordinates x, y, and z, can be written in the following form:

$$\nabla^2 c = \frac{\partial^2 c}{\partial x^2} + \frac{\partial^2 c}{\partial y^2} + \frac{\partial^2 c}{\partial z^2}\,. \qquad \text{(S2)}$$

Equation (S1) is a balance equation for ClO_2 where the two terms on the right hand side stand, successively, for the effect of the chemical reactions and of diffusional transport [1].

A simplified RD equation for ClO_2. The effective substrate concentration

As it was discussed previously there are four different amino acids and amino acid residues which can react with ClO_2 rapidly. In living tissue, however, there are even more chemical components [2] which are also able to react with ClO_2 by a slower but still measurable rate. A simple model cannot deal with all the ClO_2 reducing substrates of a complex biological system individually. To simplify the model the concept of the effective substrate concentration s will be introduced, which represents the local ClO_2 reducing capacity of all the various substrates in an integrated form.

To develop a definition for s, let us write the stoichiometry of the i-th reaction (the reaction of the i-th substrate S_i with ClO_2) in the following simplified form:

$$\nu_i \cdot ClO_2 + S_i \rightarrow \text{inert products} . \qquad (R1)$$

The stoichiometric coefficient ν_i shows how many ClO_2 moles can be reduced by one mole of S_i. For example, when the substrate contains an SH (sulfhydryl or thiol) group as cysteine does, the stoichiometric equation around pH 7 for a fast initial reaction [3] can be written as

$$2\ ClO_2 + 2\ CSH \rightarrow CSSC + 2\ ClO_2^- + 2\ H^+ \ , \qquad (R2)$$

where CSH stands for cysteine and CSSC is its oxidation product, a disulfide, called cystine. (One of the products, ClO_2^- (chlorite) is actually an intermediate because it can react further with cysteine, but only with a rate which is 6 orders of magnitude slower than the first step of the ClO_2/CSH reaction [3].) Thus, if we regard only the fast initial reaction then $\nu_{CSH} = 1$, because 1 mole CSH removes 1 mole ClO_2 in (R2). For tyrosine [4] and tryptophane [5], a simplified scheme would suggest $\nu_{TYR} = \nu_{TRP} = 2$. Even in these relatively simple cases of pure amino acids, however, the effect of various parallel and consecutive reactions [3,4,5] can make it rather difficult to calculate ν very precisely, not to mention when these amino acids are residues in proteins or peptides.

For the definition of *s*, however, it is enough to assume that there is such a stoichiometric coefficient for each component. Then *s*, the effective substrate concentration of the medium, can be defined as a weighted sum of the individual s_i substrate concentrations, where ν_i plays the role of a „weight factor":

$$s = \sum_{i=1}^{N} \nu_i \cdot s_i \ . \qquad (S3)$$

Moreover, as a further simplification, it will be assumed that R_i, the rate of the *i*-th reaction, follows mass action kinetics, that is the rate of ClO_2 reduction due to the *i*-th reaction can be written as a bilinear function of s_i and *c*:

$$R_i = \nu_i \cdot k_i \cdot s_i \cdot c \ , \qquad (S4)$$

where k_i is the second order rate constant of the *i*-th reaction. Next, introducing an „effective rate constant" *k* by the definition:

$$k = \frac{\sum_{i=1}^{N} \nu_i \cdot k_i \cdot s_i}{\sum_{i=1}^{N} \nu_i \cdot s_i} \ , \qquad (S5)$$

equation (S1) has the following simple form:

$$\frac{\partial c}{\partial t} = -k \cdot s \cdot c + D\nabla^2 c \ . \qquad (S6)$$

Simplified balance equations for fixed substrates

We will also assume all the substrates are fixed to the medium, and it is only the ClO_2 which is able to diffuse. This approximation is reasonable, if the RD medium is a human or an animal tissue having a cellular structure. Amino acid residues are usually parts of large protein molecules, the diffusion of which is very slow. Smaller peptides – especially glutathione – and free amino acids can diffuse but only within a cell because the outer membrane of the cell is not permeable for them. Thus, from the point of a long range transport through an animal or human tissue, even these small substrates can be regarded as fixed ones. This way, the general RD equation for a substrate

$$\frac{\partial s_i}{\partial t} = -k_i \cdot s_i \cdot c + D\nabla^2 s_i \qquad (S7)$$

can be simplified to

$$\frac{\partial s_i}{\partial t} = -k_i \cdot s_i \cdot c \qquad \text{(S8)}$$

as all $D_i = 0$. If we multiply both sides of equation (S8) with ν_i, and then summarize all such type of equations then we obtain the balance equation for the effective substrate concentration in the following simple form:

$$\frac{\partial s}{\partial t} = -k \cdot s \cdot c\,. \qquad \text{(S9)}$$

When the medium contains the very reactive SH groups in a significant concentration then it can be proven that (S6) and (S9) can be simplified further: the form of the equations remains the same but the effective rate constant can be approximated as

$$k \approx k_{SH} \qquad \text{(S10)}$$

where k_{SH} is the rate constant of the ClO_2 – SH group reaction, and the effective substrate concentration is

$$s \approx s_{SH} \qquad \text{(S11)}$$

where s_{SH} is the concentration of the sulfhydryl groups in the medium.

Approximate solutions of the simplified RD equations

If the simplified RD equations (S6) and (S9) are accepted as a starting point, then the logical next step is to find a solution for these equations, that is to find the functions $c=c(t,x,y,z)$ and $s=s(t,x,y,z)$ while taking into account the given initial and boundary conditions. However, to find exact analytical solutions for nonlinear partial differential equations is usually not possible, and in this work we did not want to apply numerical solutions either. Thus, our aim here should be to find and apply approximate solutions with simple mathematical formulas which can be easily applied for the interpretation of our experimental results.

One type of approximation can be applied when the rate constant *k* is very high, as in the case of substrates containing SH groups or tyrosine residues. In this case, a sharp reaction front propagates through the medium, and the solution of the reaction-diffusion problem can be approximated with „parabolic rate law" type equations.

The other approximation is valid for low *k* values. In this case, the smooth concentration profiles are determined mostly by the diffusion, and modified only slightly by the reaction which can be distributed in the whole medium (no sharp front). When the medium is finite, as in the case of a membrane, an approximate steady state can be reached after some transition time.

Quasi steady state solution of the RD equations when the ClO_2 – substrate reaction is fast

Preconditions of the parabolic rate law

The so-called parabolic rate law [6] holds for certain reaction-diffusion problems where the rate limiting step of an otherwise fast irreversible reaction (R3)

$$\mathrm{C + S \rightarrow P} \qquad \text{(R3)}$$

between the mobile reactant C and the fixed substrate S giving the product P is not the reaction itself but the diffusion of the reactant C to reach S. In our case C is ClO_2 and S is the reactive side group of an amino acid. The most important reactant in this respect is the SH group of the cysteine [3].

An important player in the process is the medium M immobilizing the substrate S but permeable for C at the same time. In our case the medium M is the hydrogel of the living tissues which is permeable for ClO_2. Lipid membranes of the cells in the tissue do not form barriers for ClO_2 either, as it is very soluble in organic phases as well. The reactive amino acids, on the other hand – being mostly building blocks of various proteins – are immobilized in that hydrogel.

The parabolic rate law in one dimension for a slab of thickness d

The simplest geometry giving a parabolic rate law is a situation where the concentration of C is kept constant, [C] = c_0 at the flat boundary of a slab e.g. at its left hand side, while [C] = 0 at the right hand side of the slab. The material of the slab is a medium containing the fixed substrate S in a homogeneous initial concentration s_0 (see Fig. S1). The thickness of the slab is *d*.

Figure S1. Schematic ClO_2 and substrate concentration profiles in a hydrogel slab of thickness *d* at an intermediate time t (0 < t < T). *p* is the penetration depth.

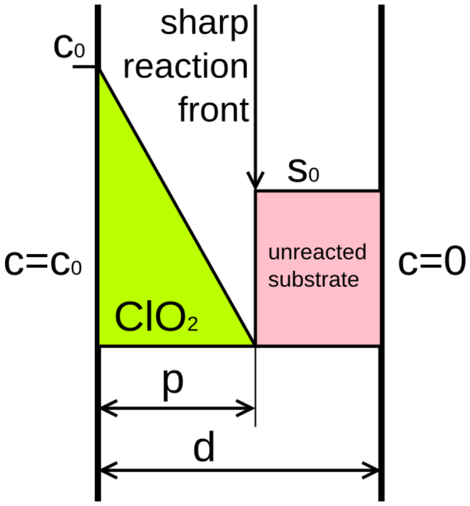

When C is ClO_2 and ClO_2 is fed from one side of the slab, a sharp reaction front propagates from one side to the other. There is a measurable ClO_2 concentration only behind the front thus disinfection of the slab is completed only when the reaction front reaches the other side of the slab. The characteristic time *T* required for that can be calculated by the parabolic rate law. The result:

$$T = \frac{s_0}{2c_0 D} d^2 \quad \textit{that is} \quad T \propto d^2 . \qquad \text{(S12)}$$

That is the characteristic time *T* is proportional with the square of the thickness for a given set of non-geometrical parameters s_0, c_0, and *D*. Here *D* is the diffusion coefficient of C in M. Alternatively, the penetration depth *p* of a sharp reaction is proportional with the square root of the time *t*:

$$p = \sqrt{\frac{2c_0 D}{s_0}} \cdot \sqrt{t} \quad \textit{that is} \quad p \propto \sqrt{t} . \qquad \text{(S13)}$$

Naturally the above formula gives the right *p* value only when $t \le T$ or when *d* is infinitely long.

Derivation of the parabolic rate law for a slab (or membrane)

As the concentration profile in Fig. S1 shows, it is assumed that the reaction occurs only in the plane $x = p$. This can be a good approximation if most of the reaction takes place in a narrow reaction zone much thinner than d (which is valid for a fast reaction combined with a relatively slow diffusion).
The ClO_2 current I_C across the slab with cross-section A in the region $0 < x < p$ can be given by Fick's law of diffusion:

$$I_C = -A \cdot D \cdot \frac{dc}{dx} \qquad \text{(S14)}$$

(I_C is positive when the ClO_2 flow points from left to right in Fig. S1).
In a quasi steady state

$$\frac{\partial c}{\partial t} \approx 0 \qquad \frac{\partial c}{\partial t} = D\frac{\partial^2 c}{\partial x^2} \quad \Rightarrow \quad \frac{\partial^2 c}{\partial x^2} \approx 0 \quad \Rightarrow \quad \frac{\partial c}{\partial x} = const \qquad \text{(S15)}$$

we can assume a linear concentration profile and so

$$I_C \approx A \cdot D \cdot \frac{c_0}{p}. \qquad \text{(S16)}$$

Next we can apply the component balance. If N_S is the mole number of the remaining S molecules in the volume V ($N_S = s_0V$) then we can write

$$\begin{aligned}
&\frac{dN_S}{dt} = \frac{d(s_0V)}{dt} = \frac{d\left(s_0A(d-p)\right)}{dt} = -I_C \\
&-s_0A\frac{dp}{dt} = -A \cdot D \cdot \frac{c_0}{p} \quad \Rightarrow \quad p\frac{dp}{dt} = D \cdot \frac{c_0}{s_0} \\
&\int_0^d p\,dp = D \cdot \frac{c_0}{s_0}\int_0^T dt \quad \Rightarrow \quad \frac{d^2}{2} = D \cdot \frac{c_0}{s_0} \cdot T \quad \Rightarrow \\
&\Rightarrow \quad T = \frac{s_0}{2c_0D} \cdot d^2
\end{aligned} \qquad \text{(S17)}$$

which is the parabolic rate law in one dimension for a slab.

The parabolic rate law for an infinitely long cylinder of radius R

In this case the characteristic time T is when the sharp reaction front starting from the surface propagating inward reaches the symmetry axis of the cylinder.

$$T = \frac{s_0}{4c_0D}R^2 \quad \text{that is} \quad T \propto R^2 \qquad \text{(S18)}$$

We shall regard concentration distributions with cylindrical symmetry where the local concentration c is a function of the radius r only – that is $c=c(r)$ – and independent of the azimuthal angle φ and the height z. In an analogy to the one dimensional case

$$I_C = -A \cdot D \cdot \frac{dc}{dr} = -2r\pi H \cdot D \cdot \frac{dc}{dr} \qquad \text{(S19)}$$

where H is the height of the cylinder. We will assume a quasi steady state concentration in the zone of $R > r > R-p$ where p is the penetration depth. If I_C is independent of r in this region then $r \cdot \frac{dc}{dr} = const$. Regarding the boundary conditions: $c(R-p) = 0$ and $c(R) = c_0$ the steady state concentration profile in this region can be written as:

$$c = c_0 \cdot \frac{ln\left(\frac{r}{R-p}\right)}{ln\left(\frac{R}{R-p}\right)} \qquad \text{(S20)}$$

and

$$\frac{dc}{dr} = \frac{1}{r} \cdot \frac{c_0}{ln\left(\frac{R}{R-p}\right)}. \qquad \text{(S21)}$$

Thus, with the above quasi steady state approximation

$$I_C = -2r\pi H \cdot D \cdot \frac{dc}{dr} \approx -2\pi H \cdot D \cdot \frac{c_0}{\ln\left(\frac{R}{R-p}\right)}. \qquad \text{(S22)}$$

The negative sign shows that I_C points inward: it is negative when $\frac{dc}{dr} > 0$.

Next we can apply the component balance. If N_S is the mole number of the remaining S molecules in the volume V ($N_S = s_0V$) then we can write

$$\frac{dN_S}{dt} = \frac{d(s_0V)}{dt} = \frac{d\left(s_0 H (R-p)^2 \pi\right)}{dt} = -|I_C|$$

$$-s_0 H\pi 2(R-p)\frac{dp}{dt} = -2\pi H \cdot D \cdot \frac{c_0}{\ln\left(\frac{R}{R-p}\right)} \quad \Rightarrow \quad (R-p)\ln\left(\frac{R}{R-p}\right)\frac{dp}{dt} = D \cdot \frac{c_0}{s_0} \qquad \text{(S23)}$$

$$\int_0^R (R-p)\ln\left(\frac{R}{R-p}\right)dp = D \cdot \frac{c_0}{s_0}\int_0^T dt \quad \Rightarrow \quad \frac{R^2}{4} = D \cdot \frac{c_0}{s_0} \cdot T \quad \Rightarrow$$

$$\Rightarrow \quad T = \frac{s_0}{4c_0 D} \cdot R^2$$

The parabolic rate law in three dimensions for a sphere of radius R

$$T = \frac{s_0}{6c_0 D} R^2 \quad \text{that is} \quad T \propto R^2 \qquad \text{(S24)}$$

We shall regard concentration distributions with spherical symmetry where the local concentration c is a function of the radius r only – that is $c=c(r)$ – and independent of the azimuthal angle φ and the polar angle Θ. In an analogy to the one dimensional case,

$$I_C = -A \cdot D \cdot \frac{dc}{dr} = -4r^2\pi \cdot D \cdot \frac{dc}{dr}. \qquad \text{(S25)}$$

We will assume a quasi steady state concentration in the zone of $R > r > R–p$ where p is the penetration depth. If I_C is independent of r in this region then $r^2 \cdot \frac{dc}{dr} = const$. Regarding the boundary conditions: $c(R–p) = 0$ and $c(R) = c_0$ the steady state concentration profile in this region can be written as:

$$c = c_0 \frac{R}{p}\left(1 - \frac{R-p}{r}\right) \qquad \text{(S26)}$$

and

$$\frac{dc}{dr} = c_0 \frac{R}{p} \cdot \frac{R-p}{r^2}. \qquad \text{(S27)}$$

Then with the quasi steady state approximation:

$$I_C = -4r^2\pi \cdot D \cdot \frac{dc}{dr} \approx -4\pi \cdot D \cdot c_0 \frac{R}{p} \cdot (R-p) \qquad \text{(S28)}$$

The negative sign shows again that I_C points inward: it is negative when $\frac{dc}{dr}>0$.

Next we can apply the component balance. If N_S is the mole number of the remaining S molecules in the volume V ($N_S = s_0 V$), then we can write

$$\frac{dN_S}{dt} = \frac{d(s_0 V)}{dt} = \frac{d\left(s_0 4(R-p)^3 \pi/3\right)}{dt} = -\left|I_C\right|$$

$$-s_0 \pi 4(R-p)^2 \frac{dp}{dt} = -4\pi \cdot D \cdot c_0 \frac{R}{p} \cdot (R-p) \quad \Rightarrow \quad \frac{p(R-p)}{R}\frac{dp}{dt} = D \cdot \frac{c_0}{s_0}$$

$$\int_0^R \frac{p(R-p)}{R} dp = D \cdot \frac{c_0}{s_0} \int_0^T dt \quad \Rightarrow \quad \frac{R^2}{6} = D \cdot \frac{c_0}{s_0} \cdot T \quad \Rightarrow \qquad \text{(S29)}$$

$$\Rightarrow \quad T = \frac{s_0}{6c_0 D} \cdot R^2$$

Quasi steady state solution of the RD equation in one dimension when the ClO_2 – substrate reaction is slow

The one dimensional reaction-diffusion equation (RDE) in a steady state is

$$0 = -r + D\frac{d^2c}{dx^2} \qquad \text{(S30)}$$

where r is the rate of the reaction (R4)

$$C + S2 \rightarrow P2 \qquad \text{(R4)}$$

between the mobile reactant C and the fixed substrate S2 giving the product P2

$$r = k_2 \cdot c \cdot s_2 . \qquad \text{(S31)}$$

In this case, however, it will be assumed that the rate of the reaction is relatively slow and the substrate S2 is in such a great excess that its consumption can be neglected during the time of the measurement. That is

$$r \approx k_2 \cdot c \cdot (s_2)_0 , \qquad \text{(S32)}$$

and the steady state RDE:

$$\frac{d^2c}{dx^2} = \frac{k_2 \cdot (s_2)_0}{D} \cdot c . \qquad \text{(S33)}$$

If we introduce the following notation

$$\frac{k_2 \cdot (s_2)_0}{D} \equiv k^2$$

then the steady state RDE has the following form:

$$\frac{d^2c}{dx^2} = k^2 \cdot c . \qquad \text{(S34)}$$

The general solution of the above differential equation is

$$c = c_1 \cdot e^{+k\cdot x} + c_2 \cdot e^{-k\cdot x} \qquad \text{(S35)}$$

where the integration constants c_1 and c_2 can be calculated from the boundary conditions:

$$c(0) = c_0 \qquad and \qquad c(d) = 0.$$

The solution regarding the above boundary conditions:

$$c = c_0 \frac{sh(k \cdot (d - x))}{sh(k \cdot d)} . \qquad \text{(S36)}$$

If the reaction is very slow, then the approximation

$$sh(\alpha) \approx \alpha \quad if \quad \alpha << 1 \qquad \text{(S37)}$$

can be applied and the steady state concentration profile obtained this way is the linear concentration profile valid for pure diffusion in the absence of any chemical reaction:

$$c = c_0\left(1 - \frac{x}{d}\right) . \qquad \text{(S38)}$$

The current density j of component C leaving the slab is maximal when there is no chemical reaction:

$$j_{MAX} = -D\frac{dc}{dx} = D\frac{c_0}{d}. \qquad \text{(S39)}$$

The current density is smaller if there is a slow reaction in the slab:

$$j_{RD} = -D\left(\frac{dc}{dx}\right)_{x=d} = D \cdot c_0 \frac{k}{sh(kd)}. \quad \text{(S40)}$$

Finally, the ratio of the current densities is:

$$\frac{j_{RD}}{j_{MAX}} = \frac{kd}{sh(kd)}. \qquad \text{(S41)}$$

Table S1

Data depicted in Figure 2.

V is the cumulative volume of the 0.01 M $Na_2S_2O_3$ titrant added until time t.

1st exp	2nd exp	
t / s	t / s	V / ml
0	0	0
540	140	0.5
630	202	1
695	252	1.5
752	294	2
809	326	2.5
855	366	3
876	403	3.5
915	440	4
955	476	4.5
995	507	5
1038	542	5.5
1078	575	6
1118	608	6.5
1153	642	7
1192	675	7.5
1226	708	8
1262	747	8.5
1299	782	9
1342	814	9.5
1374	847	10
1412	880	10.5
1450	908	11
1480	940	11.5
1518	972	12
1552	1004	12.5
1588	1038	13

Table S2
Data depicted in Figure 3.
V is the cumulative volume of the 0.01 M $Na_2S_2O_3$ titrant added until time t.

1st day	2nd day	3rd day	
t / s	t / s	t / s	V / ml
0	0	0	0
2490	530	250	0.5
2894	655	346	1
3198	750	418	1.5
3456	837	493	2
3670	916	568	2.5
3882	991	639	3
4080	1069	713	3.5
4273	1140	777	4
4452	1216	856	4.5
4636	1285	928	5
4812	1354	1003	5.5
4993	1420	1076	6
5171	1487	1147	6.5
5344	1556	1216	7
5522	1618	1286	7.5
5704	1680	1358	8
5889	1743	1427	8.5
6075	1810	1501	9
6266	1877	1579	9.5
6472	1942	1655	10
6658	2008	1734	10.5
6852	2077	1819	11
7059	2146	1894	11.5
	2218	1978	12
	2280	2061	12.5
	2346	2144	13
		2235	13.5

Supporting Information References

[1] Nicolis G, De Wit A (2007) Reaction-diffusion systems. Scholarpedia 2(9): 1475. http://www.scholarpedia.org/article/Reaction-diffusion_systems. Accessed 2013 April 15.

[2] Tan HK, Wheeler WB, Wei CI (1987) Reaction of chlorine dioxide with amino acids and peptides: Kinetics and mutagenicity studies. Mutat Res 188: 259-266.

[3] Ison A, Odeh IN, Margerum DW (2006) Kinetics and Mechanisms of Chlorine Dioxide and Chlorite Oxidations of Cysteine and Glutathione. Inorg Chem 45: 8768-8775.

[4] Napolitano MJ, Green BJ, Nicoson JS, Margerum DW (2005) Chlorine Dioxide Oxidations of Tyrosine, *N*-Acetyltyrosine, and Dopa. Chem Res Toxicol 18: 501-508.

[5] Stewart DJ, Napolitano MJ, Bakhmutova-Albert EV, Margerum DW (2008) Kinetics and Mechanisms of Chlorine Dioxide Oxidation of Tryptophan. Inorg Chem 47: 1639-1647.

[6] House JE (2008) Inorganic Chemistry. Academic Press. 259 p. (paragraph 8.2.2 The Parabolic Rate Law)
